# Spatial distribution and insecticide resistance of *Aedes* mosquitoes in Osun State: implications for vector control

**DOI:** 10.1186/s41182-025-00845-y

**Published:** 2025-11-03

**Authors:** L. O. Busari, A. S. Babalola, Q. O. Adeshina, O. G. Dauda, Z. O. Iwalewa, G. O. Ige, G. B. Jokanola, C. T. Aroyehun, M. M. Abdulsalam, Y. O. Yusuff, R. A. Oyewusi, I. O. Awoniyi, O. A. Surakat, A. O. Adeogun, A. M. Rufai, K. A. Fasasi, M. A. Adeleke

**Affiliations:** 1 Department of Biological Sciences, Fountain University, P.M.B., 4491, Osogbo, Osun State Nigeria; 2 Parasitology and Vector Biology Unit, Osun State University, Osogbo, Osun State Nigeria; 3 Molecular Entomology and Vector Control Unit, Public Health and Epidemiology Department, Nigerian Institute of Medical Research, P.M.B., 2013, Yaba, Lagos State Nigeria; 4 Pest Management and Toxicology Unit, Department of Animal and Environmental Biology, Osun State University, Osogbo, Osun State Nigeria; 5 Department of Biochemistry, Osun State University, Osogbo, Osun State Nigeria

**Keywords:** *Aedes*, Mosquito, Insecticide, Resistance, Mapping, Osun, Vector, Synergist

## Abstract

**Background:**

*Aedes* mosquitoes are primary vectors of arboviral diseases, such as dengue, chikungunya, and Zika, posing major threats to tropical public health. Understanding their spatial distribution and resistance status is vital for sustainable control. This study investigated the mapping of breeding habitats, species composition, and insecticide susceptibility in *Aedes* populations from Osun State, Nigeria.

**Methods:**

Larval surveys across a rural community identified 36 potential habitats, of which 27.8% were positive for *Aedes* breeding. A total of 3500 larvae were collected, reared to adult stage, morphologically identified and subjected to WHO-standard insecticide bioassays.

**Results:**

Two species were identified: *Aedes aegypti* (99.3%) and *Aedes albopictus* (0.7%), with *Ae. aegypti* strongly predominant (*p* < 0.05). Mortality rates following insecticide exposure ranged from 94 to 100%. Complete susceptibility was observed for permethrin, deltamethrin, and pirimiphos-methyl, while reduced mortality (94%) against alpha-cypermethrin indicated possible emerging resistance. The mapping of larval habitats revealed clustered breeding in rural communities, portending localized risk of arboviral transmission.

**Conclusions:**

These findings highlight that while *Aedes* populations in the study area remain largely susceptible to conventional insecticides, early signals of resistance require proactive management by the state. Incorporating synergists into integrated vector control, alongside habitat surveillance and mapping, will be critical to sustaining insecticide effectiveness and reducing the burden of *Aedes*-borne diseases in Osun State and Nigeria at large.

## Background

Vector-borne diseases (VBDs) remain a significant public health concern globally. Among the mosquito vectors, *Aedes* spp. are of increasing global health challenge in the transmission of viruses causing arboviral diseases, such as Dengue, Zika, and Chikungunya fever [1]. These diseases continue to pose significant public health threats worldwide, particularly in tropical and subtropical regions [2, 3]. *Aedes aegypti* and *Aedes albopictus* have demonstrated remarkable ecological adaptability and resilience in urban and peri-urban environments [4]. In Nigeria, the burden of arboviral diseases has escalated in recent years, with recurrent outbreaks necessitating the urgent need for effective vector control strategies [5]. This could be due to its endemicity for malaria, a mosquito-borne disease (MBD) with a higher global health risk and increasing burden [6].

Chemical control through the use of insecticides, especially the use of pyrethroid-based insecticides, remains a cornerstone of *Aedes* vector management due to their rapid knockdown effect, low mammalian toxicity, and cost-effectiveness [7]. However, the widespread and often indiscriminate application of pyrethroids in both public health and agricultural sectors has led to the emergence, re-emergence, and proliferation of insecticide resistance in mosquito populations [8, 9]. *Aedes* mosquitoes have been reported to be resistant to insecticides in different West African countries including Burkina Faso, Cameroon, Senegal and Ghana [10]. In Ghana, resistance of *Aedes aegypti* to three of the classes of insecticides: pyrethroids, organochlorine and carbamates recommended by WHO for vector control have been reported[11]. Resistance mechanisms in *Aedes spp*. vary, including target-site insensitivity (e.g., knockdown resistance mutations), metabolic detoxification via upregulated enzymes, such as cytochrome P450 monooxygenases, esterases, and glutathione S-transferases, and behavioral adaptation [12, 13].

In Nigeria, studies on insecticide resistance in *Aedes* populations remain limited and geographically fragmented, with a paucity of data from many states, including Osun State in the southwest region [14, 15]. In Osun State, efforts have mainly been concentrated on the *Anopheles* mosquito for malaria control and elimination due to its higher burden. This hinders the development of locally tailored and evidence-based *Aedes* vector control interventions. Moreover, the potential of synergist compounds that inhibit metabolic resistance pathways to restore the efficacy of pyrethroids has not been adequately explored [16].

Therefore, this study aims to investigate the species and the resistance status of *Aedes spp*. to commonly used pyrethroid and organophosphate insecticides in Osun State addressing a critical knowledge gap in the state, where no prior resistance data exist for Aedes mosquitoes vector control and insecticide resistance status. By elucidating the current resistance profiles and underlying mechanisms, this research seeks to inform integrated vector management (IVM) strategies and support the rational deployment of insecticides in Nigeria’s fight against arboviral diseases.

## Methods

### Study area

**Fig. 1:**
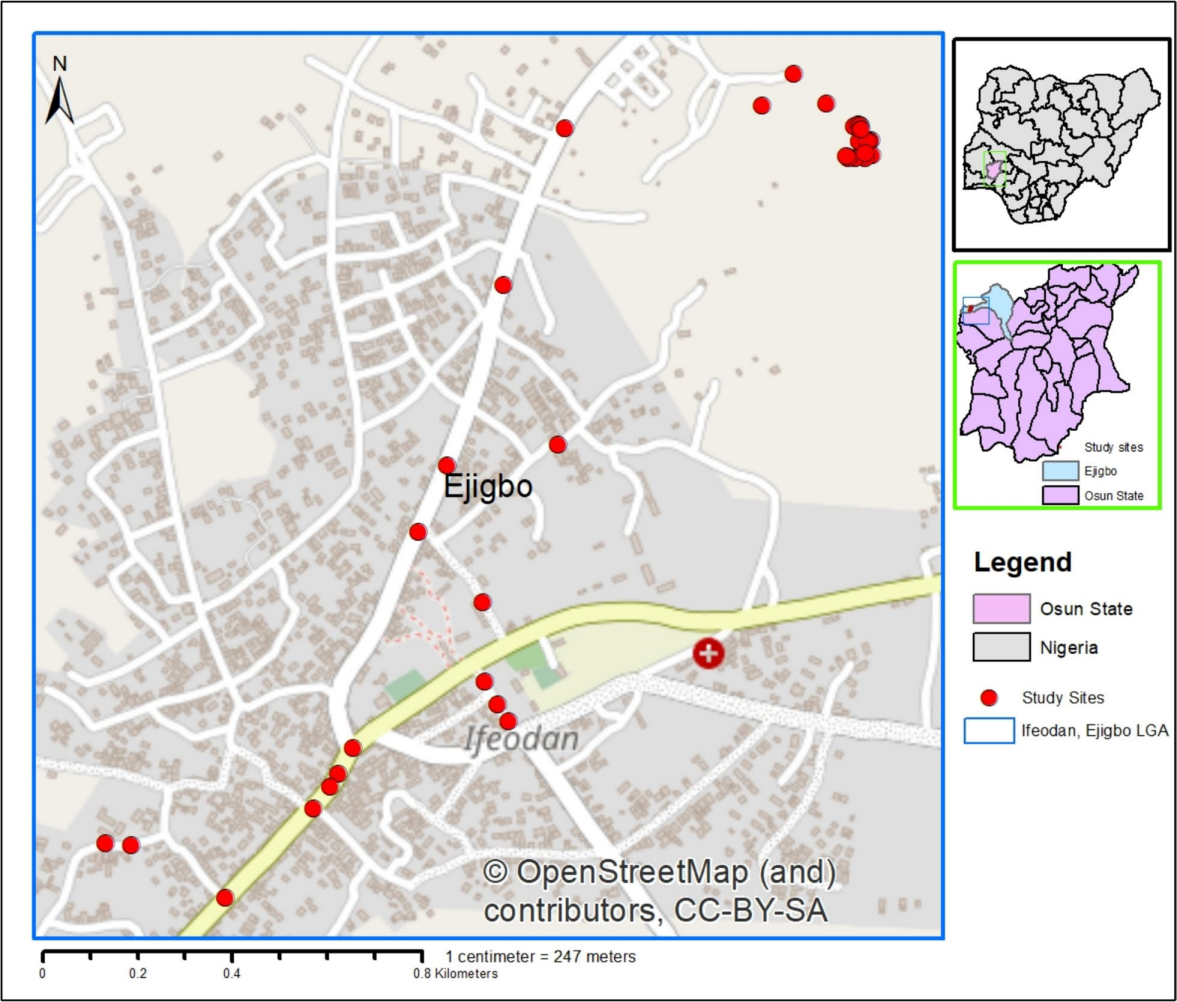
Map of the study area and sampling sites

The study was conducted at Ife-Odan (N7.833333, E4.133333), a rural area in Ejigbo Local Government (LG) of Osun State, southwest, Nigeria (Fig. 1). Previous studies have shown Ejigbo LG to be positive for larval habitat with a very high suitability for malaria mosquito vector [17]. Therefore, the selection of the study area targets the Aedes mosquitoes that transmit arboviral diseases. The study area is approximately 47.5km from Osogbo, the state capital. The major preoccupation of residents is farming and trading. However, Osun State is known for tourism and culture, attracting tourists from different parts of the world annually.

### Community entry and mobilization

Prior to the commencement of field activities, preliminary visits were conducted to the study area, to facilitate community entry and mobilization. Engagements were held with community leaders and heads of primary health centres to introduce the objectives and significance of the study. During these visits, the research team provided detailed explanations on the public health relevance of the study, particularly its potential impact on *Aedes* vector control and disease prevention.

Emphasis was placed on the broader implications for community health, with particular reference to the burden of mosquito-borne diseases such as malaria, yellow fever, lymphatic filariasis and others. The discussions aimed to build trust, encourage community participation, and ensure local support throughout the study. This approach helped to create awareness, dispel misconceptions, and promote a collaborative environment conducive to successful data collection and intervention planning.

### *Aedes* larval surveillance and collection

A comprehensive larval prospection was conducted across the study communities to identify and characterize potential *Aedes* mosquito breeding habitats. Typical breeding sites such as domestic water-holding containers, discarded plastic items, used tires, open drains, and uncovered water storage vessels were systematically surveyed. These sites were categorized into five major habitat types: gutters, ground pools, discarded containers, used tires, and open drains.

Larval sampling was carried out weekly between 07:00 and 11:00 h from January to July 2025, coinciding with peak *Aedes* larval activity. All accessible breeding sites were inspected, and immature stages (larvae and pupae) of *Aedes* spp. were collected using standard dippers, plastic scoops, and fine-mesh sieves (approximately 0.55 mm mesh size). The collected larvae were transferred into well-labeled plastic containers containing water from their respective habitats to minimize stress and mortality during transport.

Samples were then conveyed to the Molecular Epidemiology and Vector Biology Laboratory (MOVEB), Department of Animal Science and Environmental Biology, Osun State University, Osogbo, Osun State, Nigeria, for species identification and further analysis. Larvae were reared to the adult stage under controlled laboratory conditions to facilitate accurate morphological identification using standard taxonomic keys.

### Larval and adult *Aedes* mosquito identification

All collected larvae were morphologically identified to genus level using a dissecting microscope and standard taxonomic keys specific to *Aedes* spp., as described by [18]. Larvae were reared under standard laboratory conditions until they emerged as adult mosquitoes.

Emergent adult mosquitoes were transferred into species-specific holding cages and further identified to species level using morphological keys provided by [19]. Identification was based on adult morphological characteristics, such as scale patterns on the thorax, leg banding, and abdominal markings.

Confirmed *Aedes* spp. were selected for subsequent insecticide susceptibility bioassays.

### Insecticide susceptibility bioassay

Insecticide susceptibility tests were conducted following the World Health Organization (WHO) standard protocols using WHO tube bioassay kits [20]. Emergent, non-blood-fed female *Aedes* mosquitoes aged 2–3 days were selected for testing. Mosquitoes were exposed to insecticide-impregnated papers containing the following discriminating concentrations (DC): permethrin (0.75%), deltamethrin (0.05%), alpha-cypermethrin (0.05%) and pirimiphos-methyl (0.25%).

For each insecticide, four replicates of 25 female mosquitoes were tested (*n* = 100), alongside two control replicates (*n* = 50) exposed to untreated papers. Mosquitoes were introduced into exposure tubes and subjected to the insecticide-treated papers for 60 min. Knockdown was recorded at 10-min intervals throughout the exposure period. After exposure, mosquitoes were transferred to clean holding tubes and provided with 10% sugar solution. Final mortality was recorded 24 h post-exposure.

The percentage mortality was calculated using the formula:

$${\text{Percentage mortality}} = \frac{Number\,of\,tested\,female\,mosquitoes\,dead}{{Total\,number\,of\,tested\,female\,mosquitoes}} \times {1}00$$

Susceptibility status was interpreted based on WHO criteria [20]: ≥ 98% mortality: susceptible population, 90–97% mortality: possible resistance and < 90% mortality: confirmed resistance.

### Data analysis

Data obtained from the insecticide susceptibility bioassays were subjected to statistical analysis to evaluate the variation in mortality rates across different insecticides in the study location. Percentage mortality for each insecticide treatment was calculated and classified according to WHO susceptibility criteria [20], where: mortality ≥ 98% indicates full susceptibility, mortality between 90 and 97% suggests possible resistance, and mortality < 90% confirms resistance.

Descriptive statistics were used to summarize mosquito mortality rates for each insecticide treatment. Mortality percentages and their corresponding 95% confidence intervals (CIs) were calculated using the binomial exact method to account for the fixed bioassay sample size (*N* = 100).

To determine whether there were statistically significant differences in mortality rates among the insecticides and species composition, a chi-square test for proportions was employed. A significance threshold of *p* < 0.05 at a 95% confidence interval was adopted. Statistical analyses were performed using IBM SPSS Statistics, Version 21.

## Results

### *Aedes* mosquito species composition

**Fig. 2:**
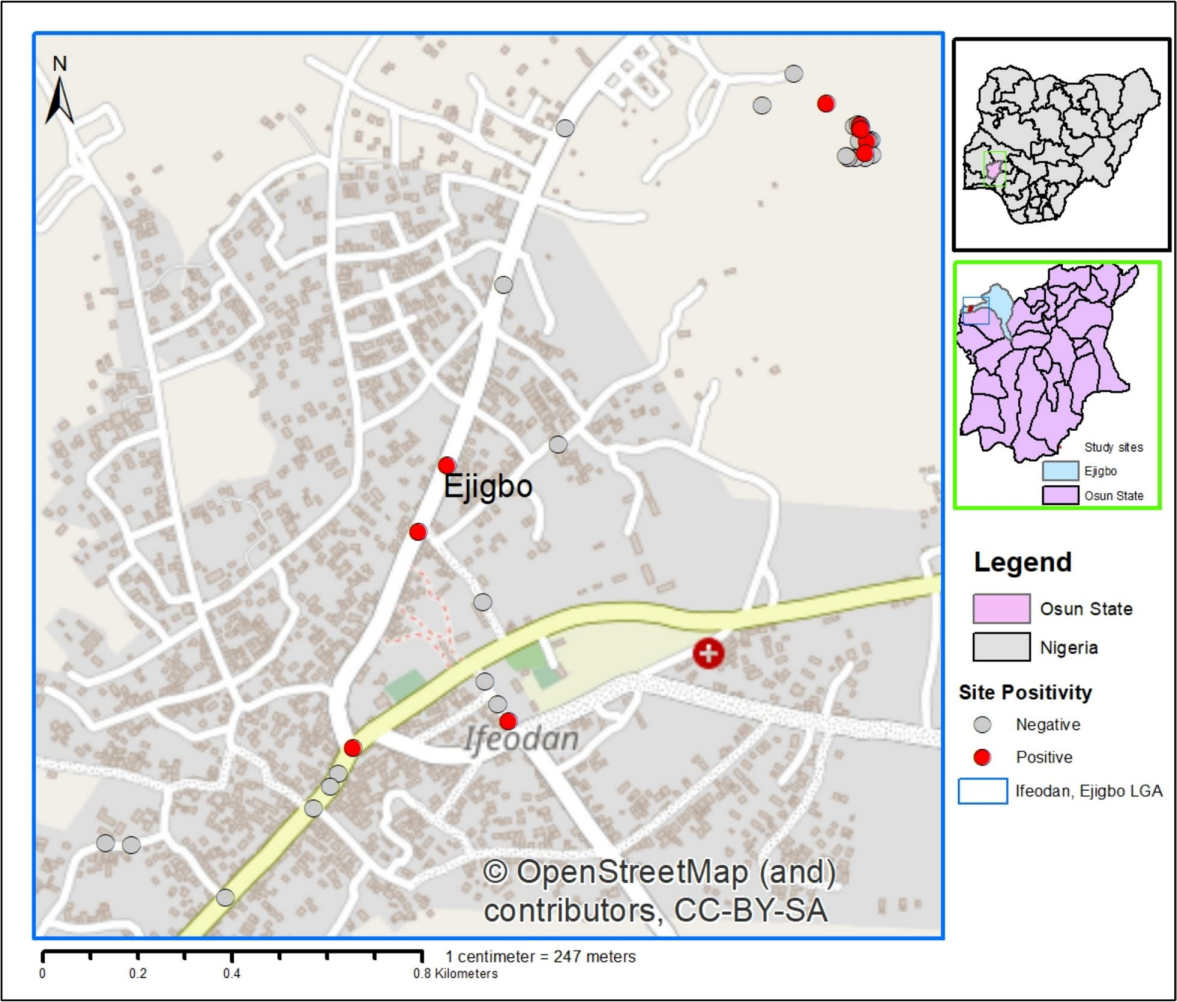
Larval sites positivity across the study area

Out of the 36 potential breeding sites examined, ten (27.8%) were found to be positive for *Aedes* mosquitoes (Fig. 2). The findings further revealed that six (60%) of these ten positive sites were clustered within less densely populated core rural areas (Fig. 2). A total of 3500 *Aedes* mosquito larvae were collected during the study period. However, out of the emergent 600 adult *Aedes* mosquito vectors, only two species were morphologically identified among the population: *Aedes aegypti* and *Aedes albopictus*. *Aedes aegypti* was predominant, 596 (99.3%), with a minimal number of *Aedes albopictus* 4 (0.7%) (Table 1). The variation in the species composition was statistically significant, showing a strong predominance of *Aedes aegypti* (*p* = 0.001; *p* < 0.05).

**Table 1:** *Aedes* mosquito species composition

Species	Number identified (*N*)	Percentage (%)
*Aedes aegypti*	596	99.3
*Aedes albopictus*	4	0.7
Total	600	100

### Mortality of adult female *Aedes* mosquitoes after insecticide exposure

**Fig. 3:**
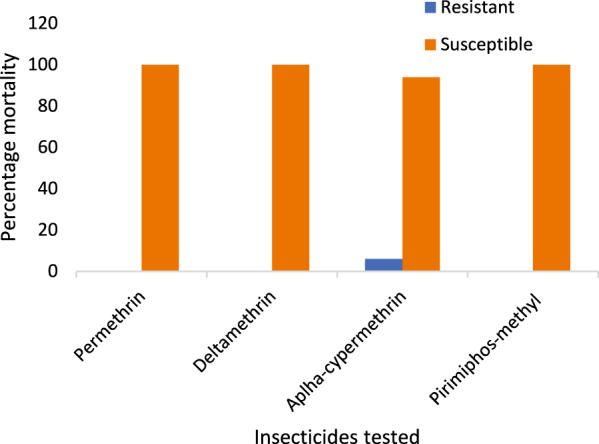
Post-exposure variation in resistance and susceptibility of *Aedes* mosquitoes to the insecticides

A total of 400 *Aedes* mosquitoes were exposed to four insecticide treatments. Overall mortality rates ranged from 94 to 100% (Table 2). After 24 h post-exposure to various insecticide treatments, differential mortality was observed among adult female *Aedes* mosquitoes, indicating varying levels of susceptibility and potential resistance (Fig. 3). The mosquitoes were all susceptible to all the tested insecticides, except in alpha-cypermethrin, where 6 (6%) resistant mosquitoes were recorded after 24 h postexposure (Fig. 3). Complete mortality (100%) was recorded for all pyrethroid-based insecticides except alpha-cypermethrin, which recorded a mortality rate of 94%, suggesting possible resistance within the population.

**Table 2:** Post-exposure of the adult female *Aedes* mosquito to the insecticides

Insecticides	Class of insecticide	Number of tested mosquitoes	Mortality (%)	95% CI	Resistance status
Permethrin	Pyrethroid	100	100	100–100	S
Deltamethrin		100	100	100–100	S
Alpha-cypermethrin		100	94	89.34–98.65	R*
Pirimiphos-methyl	Organophosphate	100	100	100–100	S

Susceptibility criteria: ≥ 98% Mortality = Susceptibility (S); 90–97% Mortality = Possible resistance (R*), *CI* = confidence interval

Percentages (%) were calculated relative to the 100 adult female mosquitoes exposed in each assay

Similarly, exposure to the organophosphate class of insecticide, pirimiphos-methyl, resulted in 100% mortality, indicating susceptibility.

Overall, the tested mosquito population exhibited high susceptibility to all evaluated insecticide classes, except for alpha-cypermethrin (*p* = 0.03; *p* < 0.05), where reduced mortality indicated an emerging resistance phenotype (Table 2).

## Discussion

The public health implications of *Aedes* mosquitoes remain profound, particularly due to their role as efficient vectors in the transmission of dengue, chikungunya, Zika, and yellow fever. The persistence of *Aedes*-borne diseases continues to challenge global vector control efforts, especially where the emergence of insecticide resistance threatens the sustainability of chemical-based interventions [20]. In this study, *Aedes* larvae were detected in 27.8% of surveyed breeding sites, with a notable concentration in rural communities. This finding supports recent evidence of *Aedes* spp. adaptability to small, transient water bodies and vegetated environments, which provide favourable microhabitats for resting and oviposition [21]. Their ecological plasticity enhances their vectorial capacity and portending the elevated risk of arboviral outbreaks in underserved rural populations across West Africa [22]. Strengthened entomological surveillance and larval source management are, therefore, essential components of integrated vector control strategies aimed at reducing *Aedes* densities and mitigating disease transmission [23].

The present study revealed a pronounced predominance of *Aedes aegypti*, accounting for 99.3% of the sampled population, while *Aedes albopictus* was observed only in minimal numbers. This strong predominance aligns with findings from Lagos State, Nigeria [24] and Ghana [25], suggesting that *Aedes aegypti* maintains an ecological advantage in urban and peri-urban habitats typical of West Africa. The detection of *Aedes aegypti* in rural settings, as observed in this study, may indicate an ongoing ecological expansion. In contrast, studies in Benin have reported a more balanced coexistence of both species [26], highlighting the influence of localized environmental and socio-ecological factors on species distribution. Given the geographical proximity of Benin to southwestern Nigeria, the potential for cross-border vector migration and expansion is a significant concern. Such transboundary dynamics necessitate coordinated regional surveillance and harmonized intervention strategies to prevent shifts in species composition that could alter arbovirus transmission patterns [22].

Insecticide susceptibility testing revealed that local *Aedes* populations remain largely susceptible to the insecticides tested, with the exception of alpha-cypermethrin, where emerging resistance was observed. This finding corroborates recent reports from Kaduna State, Nigeria, indicating resistance to alpha-cypermethrin among *Aedes* populations [27]. While earlier studies in southwestern Nigeria reported high susceptibility to conventional insecticides, the present results suggest a potential shift in resistance dynamics. Notably, these findings diverge from other regional studies that have documented widespread pyrethroid resistance [2, 25, 28]. Such inconsistencies may reflect geographical variation in insecticide exposure, differences in operational vector control practices, or climatic factors that influence selection pressures. Recent evidence suggests that climate change may play a role in modulating insecticide resistance by altering breeding site suitability and vector physiology [29]. It is plausible that localized climatic conditions in the study area have downregulated resistance gene expression, contributing to the observed susceptibility.

Although *Aedes albopictus* was detected in low numbers, its known adaptability and potential for ecological expansion warrant close monitoring. The risk of cross-border vector movement further emphasizes the need for robust surveillance systems, regional data sharing, and harmonized control strategies to mitigate the threat of arbovirus outbreaks across West Africa.

In general, these findings emphasize the continued vulnerability of *Aedes aegypti* populations in the study area to insecticide-based control measures while highlighting early warning signals of evolving resistance. The sympatric breeding of *Aedes albopictus*, albeit in low numbers, warrants attention due to its adaptability and potential to expand under favourable conditions. The risk of cross-border vector movement further stresses the importance of robust surveillance systems, regional data sharing, and harmonized control strategies to mitigate the threat of arbovirus outbreaks.

## Conclusion

The present study provides critical evidence of spatial distribution, species composition and insecticide susceptibility patterns among *Aedes* mosquitoes in the study area, reinforcing the need for proactive resistance management and integrated vector surveillance. Although high susceptibility was observed overall, the detection of reduced mortality to alpha-cypermethrin suggests early signs of possible resistance, warranting continued vigilance. Sustained monitoring through environmental management, integrated vector management (IVM), and the strategic deployment of synergists such as PBO should be prioritized. Specific recommendations include the incorporation of PBO-based nets where appropriate, integration of *Aedes* surveillance into existing malaria control programs, and the establishment of cross-border resistance monitoring initiatives. These measures are essential to preserve the long-term efficacy of vector control tools and curtail the transmission risk of *Aedes*-borne arboviral diseases in Osun State and across Nigeria.

## Data Availability

All data generated and analyzed during the study are present in the article.

## Abbreviations

PBO: Piperonyl butoxide
VBDs: Vector-borne Diseases
MBD: Mosquito-borne Disease
IVM: Integrated Vector Management
LG: Local Government
MOVEB: Molecular Epidemiology and Vector Biology
WHO: World Health Organization
DC: Discriminating Concentration
CI: Confidence Interval
OSMoH–REC: Osun State Ministry of Health Research and Ethics Committee

## References

[1] Okemena Agbor V, Idowu ET, Fagbohun IK, Oyeniyi TA, Jimoh TR, Otubanjo AO. Molecular identification and insecticide resistance status of *Culex* mosquitoes collected from blocked drainages in Lagos State, Nigeria. Pan Afr J Life Sci. 2020;4(1):1–6.

[2] Sani A, Ibrahim M, Musa H. Pyrethroid resistance in *Aedes aegypti* in northern Nigeria: implications for dengue control. J Vector Ecol. 2024;49(1):55–63. 10.1111/jvec.12567.

[3] World Health Organization (WHO). Vector-borne diseases [Internet]. Geneva: WHO; 2020 [cited 2025 Jul 8]. Available from: https://www.who.int/news-room/fact-sheets/detail/vector-borne-diseases.

[4] Kraemer MUG, Sinka ME, Duda KA, Mylne AQN, Shearer FM, Barker CM, et al. The global distribution of the arbovirus vectors Aedes aegypti and Ae. albopictus. Elife. 2015;4:e08347. 26126267 10.7554/eLife.08347 PMC4493616

[5] Nasir IA, Shuaibu SA, Usman A, Sulaiman L, Musa A. Yellow fever outbreak in Nigeria: a wake-up call. Pan Afr Med J. 2020;35(Suppl 2):9.

[6] World Health Organization. World malaria report 2024: addressing inequity in the global malaria response. Geneva: WHO; 2024.

[7] Zaim M, Aitio A, Nakashima N. Safety of pyrethroid-treated mosquito nets. Med Vet Entomol. 2000;14(1):1–5. 10759305 10.1046/j.1365-2915.2000.00211.x

[8] Hemingway J, Hawkes NJ, McCarroll L, Ranson H. The molecular basis of insecticide resistance in mosquitoes. Insect Biochem Mol Biol. 2004;34(7):653–65. 15242706 10.1016/j.ibmb.2004.03.018

[9] Moyes CL, Vontas J, Martins AJ, Ng LC, Koou SY, Dusfour I, et al. Contemporary status of insecticide resistance in the major *Aedes* vectors of arboviruses infecting humans. PLoS Negl Trop Dis. 2017;11(7):e0005625. 28727779 10.1371/journal.pntd.0005625 PMC5518996

[10] Sene NM, Mavridis K, Ndiaye EH, Diagne CT, Gaye A, Ngom EHM, et al. Insecticide resistance status and mechanisms in *Aedes aegypti* populations from Senegal. PLoS Negl Trop Dis. 2021;15:e0009393. 33970904 10.1371/journal.pntd.0009393 PMC8136859

[11] Owusu-Asenso CM, Mingle JAA, Weetman D, Afrane YA. Spatiotemporal distribution and insecticide resistance status of *Aedes aegypti* in Ghana. Parasit Vectors. 2022;15:61. 10.1186/s13071-022-05179-w. 35183249 10.1186/s13071-022-05179-w PMC8858493

[12] Liu N. Insecticide resistance in mosquitoes: impact, mechanisms, and research directions. Annu Rev Entomol. 2015;60:537–59. 25564745 10.1146/annurev-ento-010814-020828

[13] Smith LB, Kasai S, Scott JG. Pyrethroid resistance in *Aedes aegypti* and *Aedes albopictus*: important mosquito vectors of human diseases. Pestic Biochem Physiol. 2016;133:1–12. 27742355 10.1016/j.pestbp.2016.03.005

[14] Adeleke MA, Mafiana CF, Idowu AB, Adekunle MF, Sam-Wobo SO. Mosquito larval habitats and public health implications in Abeokuta, Ogun State, Nigeria. Tanzania J Health Res. 2015;17(1):1–7. 10.4314/thrb.v10i2.14348 18846789

[15] Oduola AO, Idowu ET, Oyebola MK, Adeogun AO, Olojede JB, Otubanjo OA, et al. Evidence of carbamate resistance in urban populations of *Anopheles gambiae s.s.* mosquitoes resistant to DDT and deltamethrin insecticides in Lagos, South-Western Nigeria. Parasit Vectors. 2016;9(1):477. 22686575 10.1186/1756-3305-5-116 PMC3409038

[16] Bingham G, Strode C, Tran L, Khoa PT, Jamet HP. Can piperonyl butoxide enhance the efficacy of pyrethroids against pyrethroid-resistant *Aedes aegypti*? Trop Med Int Health. 2011;16(4):492–500. 21324051 10.1111/j.1365-3156.2010.02717.x

[17] Adeleke MA, Babalola AS, Busari LO, Surakat OA, Rufai AM, Fasasi KA, et al. Modelling species distribution of *Anopheles gambiae s.l.* in Osun State using random forest modeling approach. Sci Rep. 2025;15:16524. 10.1038/s41598-025-95001-1. 40360721 10.1038/s41598-025-95001-1 PMC12075671

[18] Hopkins GHE. Mosquitoes of the Ethiopian Region I: Larval bionomics of mosquitoes and taxonomy of culicine larvae. 2nd ed. London: British Museum; 1953.

[19] Gillet JD. *Common African mosquitoes and their medical importance* (with colour illustrations). London: W Heffer & Sons; 1972.

[20] World Health Organization. Manual for monitoring insecticide resistance in mosquito vectors and selecting appropriate interventions. Geneva: WHO; 2022.

[21] Egid BR, Coulibaly M, Dadzie SK, Koudou BG, Konan LY, Gueladio C, et al. Review of the ecology and behaviour of *Aedes aegypti* and *Aedes albopictus* in Western Africa and implications for vector control. Curr Res Parasitol Vector Borne Dis. 2021;1:100074. 10.1016/j.crpvbd.2021.100074. 10.1016/j.crpvbd.2021.100074 PMC7612875 35726222

[22] Bello SOT, Zoure AA, Ouattara AK, Ouedraogo S, Sanou R, Diallo M, et al. Geographical distribution of arboviruses, *Aedes aegypti* and *Aedes albopictus* vectors and their resistance to insecticides in Africa: a systematic review. Adv Entomol. 2024;12:249–74. 10.4236/ae.2024.124019.

[23] Lepore L, Vanlerberghe V, Verdonck K, Van Bortel W, Chanda E, Wilson AL, et al. Vector control for *Aedes aegypti* and *Aedes albopictus* mosquitoes implemented in the field in sub-Saharan Africa: a scoping review. PLoS Negl Trop Dis. 2025;19(7):e0013203. 10.1371/journal.pntd.0013203. 40632827 10.1371/journal.pntd.0013203 PMC12240363

[24] Fagbohun OA, Olayemi IK, Oyewole IO. Species composition and distribution of *Aedes* mosquitoes in Lagos State Nigeria. J Mosq Res. 2020;10(3):45–52. 10.5376/jmr.2020.10.0003.

[25] Abdulai A, Konadu A, Owusu M. Insecticide resistance in *Aedes aegypti* populations in Ghana: implications for vector control. Parasit Vectors. 2023;16(1):112. 10.1186/s13071-023-05789-4.36959596

[26] Konkon NG, Dossou Y, Koudou BG. Coexistence of *Aedes aegypti* and *Aedes albopictus* in Benin: ecological implications for arbovirus transmission. Trop Med Health. 2023;51(1):89. 10.1186/s41182-023-00567-2.

[27] Yayock JY, Abubakar M, Dogo Y. Detection of alpha-cypermethrin resistance in *Aedes aegypti* populations in Kaduna State Nigeria. Afr J Infect Dis. 2022;16(1):1–9. 10.4314/ajid.v16i1.1.

[28] Pusawang K, Sattabongkot J, Saingamsook J, Zhong D, Yan G, Somboon P, et al. Insecticide susceptibility status of Anopheles and aedes mosquitoes in malaria and dengue endemic areas insects Thai-Myanmar border. Insects. 2022;13(11):1035. 36354859 10.3390/insects13111035 PMC9694411

[29] Heather M, Nicoderm A. Climate change and insecticide resistance: emerging challenges for vector control in tropical regions. Environ Health Perspect. 2024;132(2):21001. 10.1289/EHP11234.

